# Highly Stretchable, Adhesive, and Conductive PEDOT Nanocomposite Hydrogels for High‐Performance Flexible Bioelectronics

**DOI:** 10.1002/advs.202513487

**Published:** 2025-10-07

**Authors:** Huiqi Sun, Sai Wang, Peipei Wang, Ling Bai, Mingyi Tan, Fan Yang, Rongguo Wang, Xiaodong He

**Affiliations:** ^1^ National Key Laboratory of Science and Technology on Advanced Composites in Special Environments Harbin Institute of Technology Harbin 150000 China; ^2^ School of Mechatronic Engineering Shenzhen Polytechnic Shenzhen 518055 China

**Keywords:** adhesive bioelectronics, conductive hydrogel, electrophysiological signals, flexible sensors, PEDOT composite nanosheet

## Abstract

Conductive hydrogels have emerged as excellent candidates for next‐generation bioelectronic devices due to their skin‐like properties and biocompatibility. However, their practical application remains limited by poor intrinsic adhesion, insufficient electrical conductivity, and the hydrophobic nature of conventional conductive fillers, which hinder the integration of multiple essential functionalities. Herein, an effective strategy is developed to fabricate conductive and water‐soluble multifunctional nanofillers by employing tannic‐acid‐modified MXene as a template to direct the in situ polymerization of poly(3,4‐ethylenedioxythiophene) (PEDOT). The tannic acid‐modified MXene not only introduces abundant catechol groups that improve hydrophilicity and dispersibility, but also contributes intrinsic conductivity to the resulting PEDOT composite nanosheets (385 S·m^−1^). These nanosheets act as multifunctional fillers, enabling the hydrogel to simultaneously achieve excellent stretchability (>800%), strong tissue adhesion (≈22 kPa), and high conductivity (≈125 S∙m^−1^). This integrated performance exceeds that of conventional PEDOT: poly(styrenesulfonate) (PSS) based conductive hydrogels, supporting reliable and high‐resolution acquisition of electrophysiological signals in vivo and in vitro. The proposed strategy provides an effective platform for the development of advanced soft bioelectronic materials and expands the application potential of hydrogel‐based bioelectronics.

## Graphical abstract

A multifunctional and water‐soluble conductive nanocomposite is fabricated by employing tannic acid‐modified MXene as a template to direct the in situ polymerization of poly(3,4‐ethylenedioxythiophene) (PEDOT). The resulting PEDOT nanosheets are uniformly integrated into hydrogel networks, simultaneously imparting stretchability (>800%), strong tissue adhesion (≈22 kPa), and high conductivity (≈125 S·m^−1^), enabling high‐fidelity electrophysiological signal recording in vivo and in vitro.

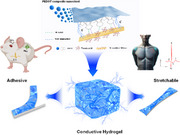

## 1 Introduction

In recent years, the rapid advancement of intelligent healthcare has driven the rapid development of bioelectronics, such as implantable devices,^[^ ^1^ ^]^ wearable sensors,^[^ ^2^, ^3^, ^4^, ^5^ ^]^ and soft actuators.^[^ ^6^ ^]^ Among various bioelectronic applications, the recording of electrophysiological signals such as electrocardiograms (ECG), electromyograms (EMG), and electroencephalograms (EEG) plays a crucial role in advancing the understanding of neural dynamics and a wide array of physiological processes.^[^ ^7^, ^8^, ^9^, ^10^, ^11^ ^]^However, conventional sensors made from silicon or metal‐based materials possess intrinsic properties such as dry, rigid, and poor biocompatibility,^[^ ^12^ ^]^ which hinder their integration with soft tissues and limit their suitability for next‐generation flexible bioelectronics. In response to these limitations, conductive hydrogels have attracted growing interest due to their skin‐like mechanical properties, such as wetness, softness, and conductivity, effectively bridging the gap between electronic systems and the human body.^[^ ^13^ ^]^ Furthermore, high conductivity is essential for bioelectronic materials to efficiently detect subtle human movements or electrophysiological signals and ensure reliable signal transmission.^[^ ^14^, ^15^ ^]^ A common and straightforward strategy to enhance conductivity is the incorporation of conductive materials such as metal nanoparticles,^[^ ^16^, ^17^, ^18^ ^]^ carbon nanotube,^[^ ^19^ ^]^ and graphene.^[^ ^20^ ^]^ However, their cytotoxicity hinders practical applications.^[^ ^21^, ^22^, ^23^ ^]^ Alternatively, hydrogels based on conducting polymers, such as poly(3,4‐ethylenedioxythiophene) (PEDOT), polypyrrole, and polyaniline, are considered as promising materials for bioelectronics, by their intrinsic superior flexibility and biocompatibility.^[^ ^24^, ^25^, ^26^, ^27^ ^]^ PEDOT has been extensively studied for its high electrical conductivity. However, its inherent hydrophobicity, stemming from its intrinsic chemical structure, poses a significant challenge for integration into a hydrogel matrix. To address this, poly(styrenesulfonate) (PSS) is commonly introduced as a dopant and dispersant to facilitate the stable dispersion of PEDOT in water. However, the presence of excess PSS significantly compromises the electrical conductivity of the resulting hydrogel.^[^ ^13^ ^]^ While a recent work reports the highest electrical conductivity (8.8 S∙cm^−1^) of pure PEDOT: PSS hydrogels,^[^ ^28^ ^]^ the hydrogel requires concentrated sulfuric acid to fabricate, and the electrical conductivity is tested in an acidic solution (pH = 1), making it unsuitable for in vivo bioelectronic applications. Therefore, ensuring stable aqueous dispersion of PEDOT as well as its effective incorporation and homogeneous distribution within the hydrogel matrix remains a significant challenge.

In addition to high conductivity, next‐generation conductive hydrogels for bioelectronic applications must also exhibit mechanical stretchability and strong adhesion to biological tissues, enabling them to accommodate dynamic physiological deformations and maintain stable signal interfaces. However, current hydrogels often face significant trade‐offs among these essential properties. Most conductive hydrogels possess poor mechanical properties and become damaged when stretched, which limits their practical applications in complex motion sensing environments.^[^ ^15^ ^]^ To address this issue, strategies such as softening the polymer network or introducing flexible components have been proposed.^[^ ^20^, ^29^, ^30^ ^]^ While these approaches can improve mechanical performance, they often dilute the conductive phase, leading to a significant reduction in conductivity (typically below 1  S∙cm^−1^).^[^ ^9^, ^31^, ^32^ ^]^ Meanwhile, efforts to enhance tissue adhesion have focused on the introduction of catechol groups and dynamic covalent bonds. Although these modifications have shown some effectiveness, they generally offer limited tunability and fail to address the requirement for high electrical performance. Notably, the synergistic integration of strong adhesion with high conductivity has yet to be fully explored.^[^ ^33^, ^34^, ^35^ ^]^ Therefore, there is an urgent need for conductive fillers capable of integrated functionality through structural design, to address the growing demands of next‐generation bioelectronics.

Herein, an effective strategy is developed by modifying MXene nanosheets with tannic acid to create a reactive and hydrophilic template (MX‐T), which facilitates the in situ polymerization of EDOT on their surfaces, leading to the formation of PEDOT composite nanosheets (MX‐T‐P) with outstanding aqueous dispersibility and electrical conductivity. The tannic modification layer increases the density of surface‐active sites density on MXene, enhancing its interaction with EDOT and promoting multiscale hydrogen bonding interactions between micron‐scale electrically conducting MXene and nano‐scale conducting polymer PEDOT for enhanced conductivity. Finally, based on the excellent dispersion of MX‐T‐P, these nanosheets were employed as nanofillers to construct conductive hydrogels integrating stretchability, adhesiveness, and conductivity. The fabricated conductive hydrogel exhibited reliable performance in capturing high‐quality electrophysiological signals, highlighting its potential for bioelectronic applications. Accordingly, this work offers a practical strategy for the design of next‐generation flexible bioelectronic materials.

## 2 Results and Discussion

### 2.1 Design Strategy

**Figure 1:**
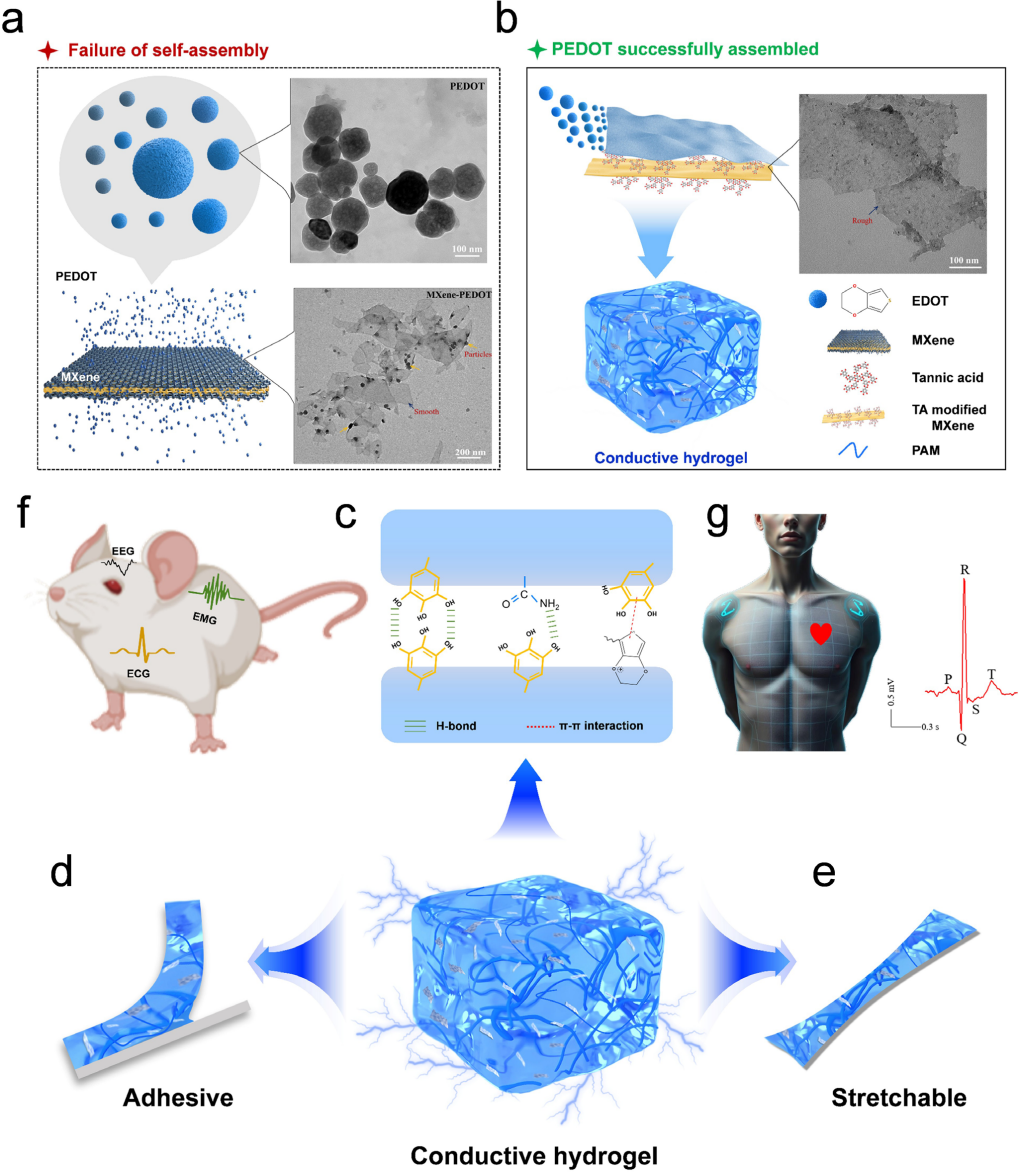
Design strategy, properties, and bioelectronic applications of PEDOT nanocomposite conductive hydrogels. a) Phase‐separated morphology of Mxene‐PEDOT. Scale bar for PEDOT: 100 nm, for MXene‐PEDOT: 200 nm. b) In situ polymerization of EDOT on MX‐T template followed by incorporation into PAM hydrogel matrix. Scale bar for MX‐T‐P: 100 nm. c) Schematic illustration of the intermolecular interactions between MX‐T‐P nanocomposites and PAM chains. d, e) The PEDOT nanocomposite conductive hydrogel exhibits self‐adhesion and stretchability. f, g) Application of the PEDOT nanocomposite conductive hydrogel in electrophysiological signal acquisition.

MX‐T‐P nanosheets were prepared by modified MXene nanosheets with tannic acid (TA) to construct a functionalized template, which subsequently guided the in situ oxidative polymerization of EDOT on its surface. MXene, owing to its abundant hydrophilic surface terminations and 2D layered structure, demonstrates excellent aqueous processability and has the potential to act as a structural template that directs the ordered alignment of PEDOT chains. However, the direct utilization of pristine MXene as a template for PEDOT polymerization encounters two principal limitations: (i) the oxidized PEDOT backbone possesses a high positive charge density (approximately one positive charge per three EDOT units), necessitating the presence of negatively charged species to maintain electrostatic neutrality,^[^ ^36^ ^]^ (ii) the inherently weak interaction between unmodified MXene and PEDOT often leads to insufficient interaction, thereby resulting in unstable composite dispersions and compromised electrical conductivity. As shown in **Figure** **1**a, the Mxene‐PEDOT composite exhibits a phase‐separated morphology. Thus, MXene must be modified with functional groups to act as a dopant of PEDOT. To address these limitations, MXene was modified with TA, a naturally derived polyphenol known for its excellent water solubility and chemical reactivity. The abundance of pyrogallol and phenolic hydroxyl groups in TA imparts strong electron‐donating and coordination capabilities, making it highly suitable for surface modification. The TA modification layer plays a dual functional role. First, increases the density of surface‐active sites on MXene, significantly enhancing its adsorption capacity for PEDOT monomers and improving the interaction between the two components. This effectively alleviates the issue of PEDOT aggregation in aqueous media. Second, the abundant phenolic hydroxyl groups in TA act as electron donors, promoting the directional oxidative polymerization of EDOT. Besides, enriched with hydrophilic functional groups such as hydroxyl and phenolic hydroxyl moieties, the nanosheets exhibit excellent aqueous dispersibility.

Based on the excellent aqueous dispersibility of MX‐T‐P nanosheets, they were successfully incorporated into the polyacrylamide (PAM) hydrogel network (Figure 1b). The nanosheets are uniformly distributed within the chemically crosslinked PAM network and interact with PAM chains through multivalent non‐covalent interactions, including hydrogen bonding, electrostatic attraction, and π–π stacking (Figure 1c). These interactions, in conjunction with the chemical crosslinking network, endow the hydrogel with enhanced stretchability. Moreover, the polyphenolic groups derived from tannic acid impart self‐adhesive properties to the hydrogel, while the π‐conjugated structure of PEDOT facilitates efficient electron transport throughout the network (Figure 1d,e). As a result, a multifunctional hydrogel with integrated stretchability, adhesiveness, and conductivity was successfully fabricated for bioelectronic applications, such as capturing high‐quality electrophysiological signals (Figure 1f,g). This work thus provides a practical strategy for the development of next‐generation flexible bioelectronic materials.

### 2.2 Characterization of PEDOT Nanocomposite

The formation of PEDOT nanocomposites was achieved by MX‐T templated‐inducted PEDOT assembly in an aqueous solution. To investigate the self‐assembly mechanism of PEDOT nanocomposites, we carried out a comprehensive morphological and structural characterization, beginning with the analysis of pristine MXene. As shown in Figure S1 (Supporting Information), the freshly prepared MXene dispersion appears black in color. Upon dilution, it transitions to a light green hue and exhibits a pronounced Tyndall effect, indicating good colloidal stability. Transmission electron microscopy (TEM) reveals that the surface of the MXene nanosheets is clean and free of noticeable oxidized particles (Figure S2a, Supporting Information). Furthermore, atomic force microscopy (AFM) measurements indicate that the nanosheets thickness of ≈1.2 nm (Figure S2b, Supporting Information), which is in agreement with previous reports.^[^ ^37^, ^38^ ^]^ In addition, X‐ray diffraction analysis further confirmed the successful synthesis of MXene (Figure S3, Supporting Information). Compared with the Ti_3_AlC_2_ precursor, the MXene shows a complete disappearance of the 39° (104) diffraction peak, along with broadening and a leftward shift of the (002) peak, indicative of the structural transformation and successful etching. Taken together, the systematic characterizations verify the successful preparation of high‐quality MXene nanosheets.

**Figure 2:**
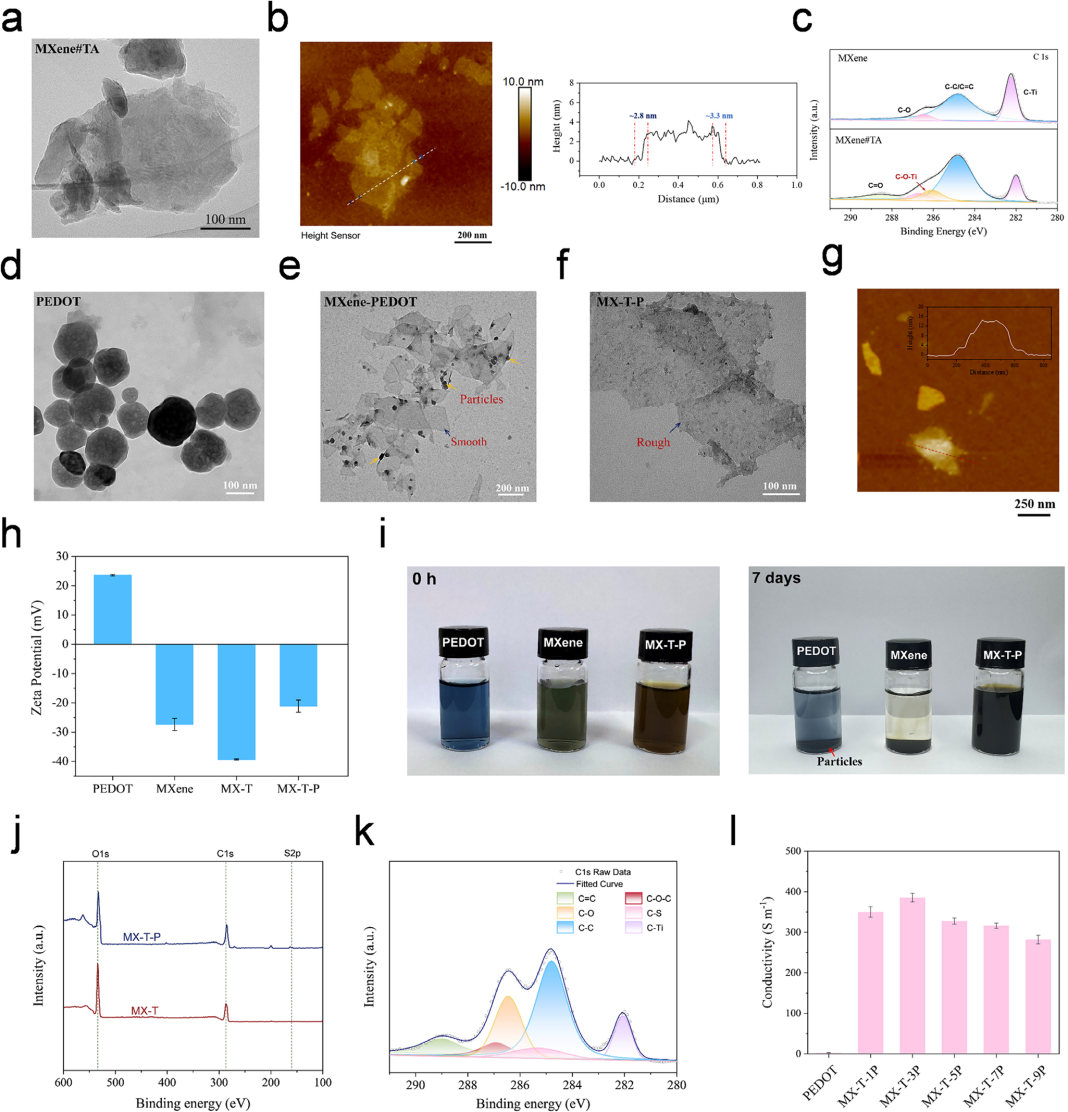
Characterization of PEDOT nanocomposites. a) TEM image of MXene#TA nanosheets dispersed in aqueous solution. b) AFM image and height profile of MXene#TA nanosheets. c) XPS spectra of C 1s for MXene and MXene#TA. d) TEM image of PEDOT synthesized via conventional oxidative polymerization. e) TEM image of MXene‐PEDOT. f) TEM image of MX‐T‐P nanocomposites. g) AFM image and height profile MX‐T‐P nanocomposites. h) Zeta potential of aqueous solutions of PEDOT, MXene, MX‐T, and MX‐T‐P. i) Dispersion of PEDOT, MXene, and MX‐T‐P nanomaterials in water. j) XPS spectra of MX‐T‐P and MX‐T. k) High‐resolution C1s XPS spectra of MX‐T‐P. l) The electrical conductivity of PEDOT and MX‐T‐P nanosheets.

Subsequently, the morphological and structural evolution upon TA modification was investigated. As seen in **Figure** **2**a, the MXene#TA maintained the typical 2D layered structure of the original MXene, indicating that the surface modification with tannic acid did not disrupt its structural integrity. AFM showed that the average thickness increased from ≈1.2 to ≈2.9 nm upon TA modification, more than a twofold increase (Figure 2b). To quantitatively assess the elemental variation introduced by TA modification, energy‐dispersive spectroscopy (EDS) was conducted at three randomly selected regions for each sample (Figures S4 and S5, Supporting Information). As summarized in Table S1 (Supporting Information), the atomic percentage of C increased markedly from 23.08% in pristine MXene to 43.58% in MXene#TA, while Ti content decreased from 51.28% to 23.53%. X‐ray photoelectron spectroscopy (XPS) provided further insights into the interfacial interactions. As shown in Figure 2c and Figure S6 (Supporting Information), the C 1s signal of MXene#TA significantly intensified. Notably, new peaks emerged at 286.07 eV (C 1s) and 532.78 eV (O 1s), which can be attributed to the interaction between phenolic hydroxyl groups of TA and surface Ti‐OH groups. These interactions indicate the formation of Ti‐C‐O‐H···O coordination structures, where phenolic groups from TA coordinate with surface Ti atoms, thereby reinforcing the interfacial interaction.

To further elucidate the critical role of MXene#TA in the assembly of PEDOT, a comparative analysis of the microscopic morphologies was conducted. In conventional oxidative polymerization,^[^ ^39^ ^]^ EDOT monomers tend to form aggregated spherical PEDOT particles, as shown in Figure 2d. When unmodified MXene nanosheets were physically mixed with EDOT (Figure 2e), the absence of strong interfacial interactions resulted in weak physical adsorption. Consequently, PEDOT particles were randomly and non‐uniformly distributed around the MXene sheets, exhibiting a clear separation between the two components. In stark contrast, the use of MX‐T as a template resulted in markedly different assembly behavior. As shown in Figure 2f, the initially smooth surface of MX‐T became noticeably rougher, indicating that EDOT polymerization occurred directly on the MX‐T surface. In line with this observation, TEM‐EDS mapping (Figure S7, Supporting Information) reveals a uniform distribution of C, O, Ti, and S across the MX‐T‐P nanosheets. AFM results further corroborated these findings. As shown in Figure S8 (Supporting Information), pristine PEDOT synthesized via conventional polymerization exhibited spherical particles with an average diameter of ≈6.7 nm. In contrast, the MX‐T‐P nanosheets obtained via template‐induced polymerization exhibit a thickness of ≈13 nm (Figure 2g), which is significantly greater than that of MX‐T. To gain deeper insight about the assembly process, zeta potentials of PEDOT, MXene, MX‐T, and MX‐T‐P were measured (Figure 2h). The unmodified MXene exhibited a zeta potential of −27.37 mV, while TA modification further reduced the potential to −39.35 mV, confirming the successful surface modification. After PEDOT assembly, the zeta potential of MX‐T‐P increased to −21.1 mV, indicating the incorporation of positively charged PEDOT chains (oxidized PEDOT exhibits a zeta potential of +13.57 mV). This shift suggests strong electrostatic interactions between the PEDOT and negatively charged MX‐T surface, which effectively drove the in situ polymerization. The negative potential of MX‐T‐P also indicates dynamic charge balancing within the system, contributing to its colloidal stability. This electrostatic stabilization is reflected in the superior aqueous dispersibility of the MX‐T‐P nanocomposites. As shown in Figure 2i, the MX‐T‐P dispersion remained stable without visible precipitation after 7 days of storage, in contrast to pristine PEDOT, which tends to aggregate due to its hydrophobicity. Similarly, PEDOT‐MXene dispersions without TA modification rapidly formed visible precipitates and large aggregates (Figure S9, Supporting Information), indicating poor dispersion stability. In addition, TEM images (Figure 2e) reveal a phase‐separated structure in unmodified PEDOT‐MXene, with PEDOT particles distributed non‐uniformly around MXene sheets. Together, these observations demonstrate that TA modification is critical for stabilizing aqueous dispersions, which is essential for forming hydrogels with continuous conductive pathways. The chemical structure of the composites was analyzed by Fourier‐transform infrared spectroscopy (FTIR) and XPS. The ATR‐FTIR spectrum of MX‐T‐P (Figure S10, Supporting Information) confirms the successful polymerization of PEDOT on the MX‐T template, as indicated by the characteristic C═C stretching of TA^[^ ^40^ ^]^ and the C─S─C stretching vibrations of the thiophene ring at 973, 915, and 572 cm^−1^.^[^ ^41^, ^42^ ^]^XPS further supported the chemical structure of the composites. The full survey spectrum (Figure 2j) displayed a distinct S 2p signal from PEDOT, while the high‐resolution C 1s spectra (Figure 2k) showed peaks at 286.6 eV (C─O(H), phenolic groups from TA) and 285.3 eV (C─S bonds). The Ti 2p spectrum (Figure S11, Supporting Information) verified the preserved MXene structure, and the S 2p region exhibited characteristic spin‐orbit split peaks of thiophene‐type sulfur.^[^ ^43^, ^44^, ^45^, ^46^ ^]^ Collectively, these results confirm the coexistence of MXene, TA, and PEDOT, and demonstrate the successful polymerization of PEDOT on MX‐T nanosheets.

The MX‐T‐P nanosheets also exhibited excellent electrical properties. As shown in Figure 2l, the electrical conductivity of MX‐T‐3P reached 385 S·m^−1^, over two orders of magnitude higher than that of pristine PEDOT synthesized via conventional solvent‐based oxidative polymerization (≈3 S·m^−1^). This remarkable enhancement can be attributed to two synergistic factors: (i) the TA modification layer increases the surface density of active sites on MXene, strengthening its interaction with EDOT and promoting multiscale hydrogen bonding between the microscale conductive MXene and the nanoscale conducting polymer, thereby facilitating the formation of more continuous conductive pathways; and (ii) the synergistic integration of PEDOT and MXene significantly enhances charge transport efficiency. However, a further increase in PEDOT nanocomposites content led to a decline in conductivity. This reduction is likely due to the excessive deposition of hydrophobic PEDOT, which masks the hydrophilic phenolic groups on the MX‐T surface, thereby weakening dispersion stability and disrupting the conductive network. Furthermore, the MX‐T‐P nanosheets exhibited stable conductivity (Figure S12, Supporting Information), which ensures long‐term electrical stability. Taken together, the assembly mechanism can be analyzed from the perspective of intermolecular interactions. The interface between PEDOT and MX‐T is stabilized by multiple non‐covalent forces. Specifically, the oxygen‐rich surface functionalities and relatively low surface potential of MX‐T enhance its interaction for EDOT, enabling more controlled polymerization and improved interfacial stability. Under mildly basic conditions, the positively charged PEDOT backbone (in its oxidized state) interacts electrostatically with the partially deprotonated phenolic hydroxyl groups of TA, thereby promoting the formation of stable self‐assembled structures. Furthermore, the polyphenol groups in TA contribute additional interfacial stability through hydrogen bonding, electrostatic interactions, and π–π stacking, which not only guide the directional polymerization of EDOT on the MX‐T surface but also impart excellent water dispersibility. Collectively, these features make it highly suitable for incorporation into hydrogel matrices, offering significant potential for applications in flexible bioelectronics.

### 2.3 Mechanical and Adhesiveness of the PEDOT Composite Hydrogel

**Figure 3:**
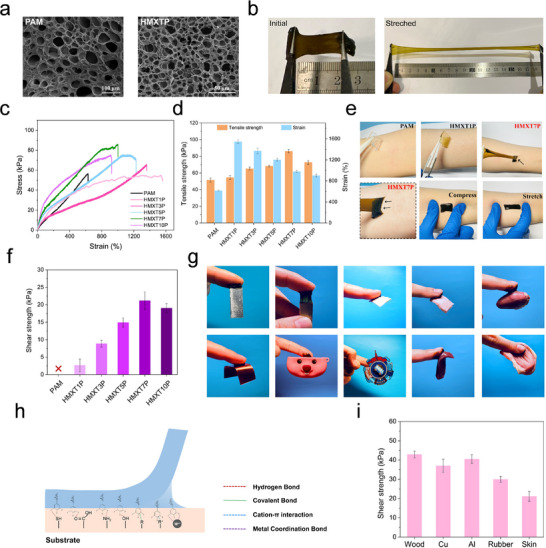
Mechanical and adhesive properties of HMXTP hydrogels. a) The micromorphology of PAM and HMXTP hydrogels. b) Photographs of HMXTP hydrogel before and after stretching at a large strain. c) and d) Mechanical properties of PAM and HMXTP hydrogels with different nanosheet contents. e) Comparative of different hydrogel‐skin adhesion interfaces. f) The summary of the shear strength of PAM and HMXTP hydrogels. g) Macroscopic images of HMXTP hydrogel adhesion on different material surfaces. h) Adhesion mechanism of HMXTP hydrogel. i) The shear strength of HMXTP hydrogels adheres to different engineering solids and biological tissues.

The MX‐T‐P nanosheets, enriched with hydrophilic functional groups such as hydroxyl and phenolic hydroxyl moieties, exhibit excellent dispersibility in aqueous environments and can be uniformly integrated into the PAM hydrogel network. These nanosheets establish multiple non‐covalent interactions with the PAM chains, such as hydrogen bonding and electrostatic interactions, which contribute to the regulation and optimization of the hydrogel's internal microstructure. Microstructure plays a pivotal role in determining the overall performance of hydrogel materials. In particularly, the optimization of the internal structure not only significantly influences the mechanical properties but also affects functional attributes such as sensing performance. As shown in **Figure** **3**a, the microstructure of the freeze‐dried HMXTP hydrogel differs significantly from that of the PAM hydrogel. As illustrated in Figure S13 (Supporting Information), the PAM hydrogel displays a large‐pore structure, with an average pore size of 56.7 µm, which is typically associated with inferior mechanical properties. Upon incorporation of MX‐T‐P nanosheets into the PAM network, the overall porous morphology is retained, but the pore size is significantly reduced to an average of 10.9 µm, and no aggregation of the nanosheets is observed. The observed reduction in pore size can be attributed to the excellent dispersibility of MX‐T‐P nanosheets and their strong interactions with the hydrogel matrix. The polyphenol of the nanosheets, mainly the phenolic hydroxyl groups from tannic acid, can form hydrogen bonds with the amide groups in PAM, thereby reinforcing the crosslinking density of the hydrogel network. The formation of such hydrogen bonds not only enhances the structural stability of the hydrogel but also increases the network density.^[^ ^47^, ^48^ ^]^ As the degree of crosslinking increases, the network becomes more compact, resulting in a noticeable reduction in pore size. The incorporation of MX‐T‐P nanosheets into the PAM hydrogel significantly enhances its toughness and stretchability. As shown in Figure 3b, the HMXTP hydrogel can withstand strains of up to ≈900% without fracturing, demonstrating its excellent stretchability. The hydrogel containing 0.7 wt.% nanosheets (HMXT7P) exhibited the best mechanical performance, achieving a high elongation of 970% and a tensile strength of 86 kPa (Figure 3c). Notably, its stiffness is comparable to that of soft biological tissues such as muscle and is ≈5 orders of magnitude lower than that of conventional metallic electrodes, making it more suitable for integration into flexible bioelectronic systems. Tensile tests showed that the tensile strength of the hydrogel initially increased and then decreased with higher MX‐T‐P nanosheet content, while the elongation at break showed a monotonic decrease (Figure 3d). Nonetheless, the overall mechanical performance remained significantly superior to that of conventional PAM hydrogels. This mechanical enhancement is attributed to the multiple reinforcement mechanisms of the MX‐T‐P nanosheets. First, the abundant hydrophilic groups on MX‐T‐P facilitate uniform dispersion within the hydrogel matrix, effectively reducing minimizing the risk of stress concentration caused by nanosheet aggregation. Second, the uniformly distributed MX‐T‐P nanosheets act as nanofillers that substantially strengthen the network. Third, the polyphenol structure of the MX‐T‐P can form extensive hydrogen bonds with the amide groups of the PAM matrix. These dynamic hydrogen bonds serve as sacrificial bonds that dissipate energy during deformation, thereby enhancing the hydrogel's toughness. To validate this mechanism, cyclic tensile loading–unloading tests were performed on PAM and HMXTP hydrogels under gradually increasing strain. As shown in Figure S14 (Supporting Information), the HMXTP hydrogels exhibited larger hysteresis loops and greater energy dissipation than PAM hydrogels, confirming their superior ability to dissipate deformation energy through a denser network and abundant dynamic hydrogen bonding interactions. In addition to these outstanding properties, the HMXTP hydrogels maintain their key mechanical properties and demonstrate good fatigue resistance under repeated deformations (Figure S15, Supporting Information), which is essential for long‐term bioelectronic applications.

In bioelectronic applications such as epidermal sensors and implantable devices, hydrogel adhesion plays a vital role. This strong adhesion is critical for ensuring consistent and long‐term acquisition and transmission of bioelectrical signals. A conformal and stable adhesive interface ensures efficient bidirectional signal transduction. As shown in Figure 3e, the PAM hydrogel showed negligible adhesion. HMXT1P exhibited modest adherence, which was easily debonded under slight force. In contrast, HMXT7P exhibited strong adhesion, forming a serrated interfacial morphology and maintaining stable contact even under large deformation. Notably, it maintained conformal contact with the skin under both compressive and tensile strain, demonstrating excellent adhesive stability during dynamic body movements. To quantitatively evaluate adhesion ability, shear adhesion tests were performed (porcine skin as a model for human skin). As shown in Figure 3f, the adhesion strength was dependent on the PEDOT composite nanosheet content. The HMXT7P hydrogel exhibited the highest adhesion strength. However, a further increase in nanosheet concentration led to a decline in adhesion performance. This trend is consistent with mechanical testing results, where excessive nanosheet content induced over‐crosslinking, reducing the hydrogel's energy dissipation capacity and increasing rigidity. As shown in Figure 3g,i, the HMXT7P hydrogel can also adhere to various materials, including wood, rubber, metal surfaces, and biological tissues. The observed adhesive behavior is primarily attributed to the synergistic effect of multiple physical and chemical interactions at the hydrogel‐substrate interface. Specifically, polyphenolic groups derived from tannic acid participate in hydrogen bonding, coordination interactions, and cation‐π interactions with diverse surfaces, thereby conferring intrinsic self‐adhesive properties to the hydrogel (Figure 3h). In contrast, the pristine PAM hydrogel, which lacks these functional groups, exhibits negligible adhesion. As for biological tissue, the adhesion process involves interactions with active functional groups, such as carboxyl, amine, and thiol groups.^[^ ^49^ ^]^ During the initial adhesion phase, phenolic hydroxyl groups rapidly form hydrogen bonds and electrostatic interactions with amine and thiol groups in the hydrated environment, enabling immediate attachment. Over time, these non‐covalent interactions are further strengthened by covalent bond formation through Schiff base or Michael addition reactions with tissue amines or thiols, thereby facilitating durable and stable interfacial adhesion.^[^ ^50^, ^51^, ^52^ ^]^ In addition, the HMXTP hydrogel also shows long‐term and repeatable adhesiveness (Figure S16, Supporting Information) without causing skin irritation or leaving residue, highlighting its potential for bioelectronic applications.

### 2.4 Application of PEDOT Composite Hydrogels in Bioelectrical Signal Monitoring

**Figure 4:**
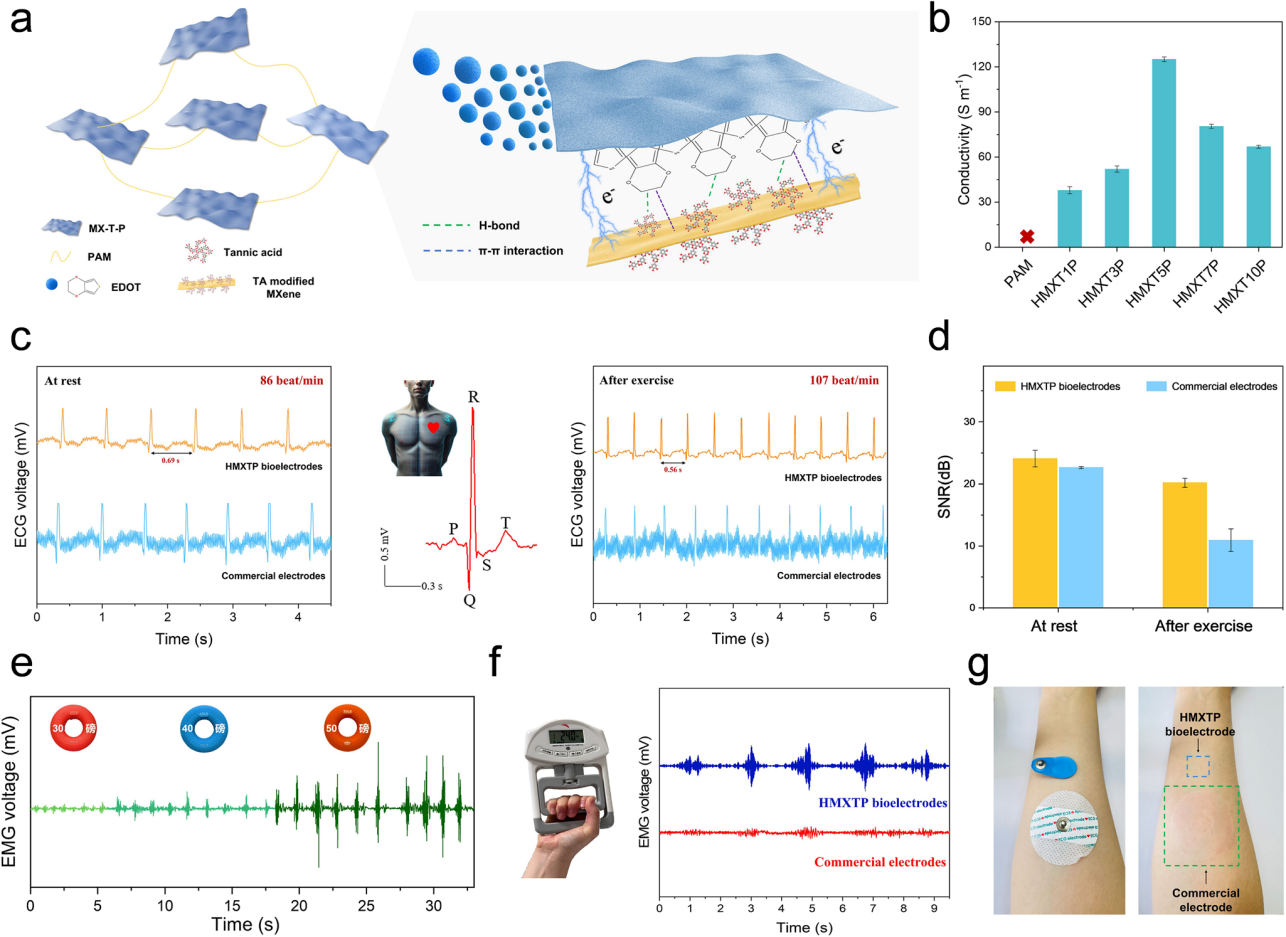
Conductivity and bioelectrical signal recording performance of the HMXTP hydrogels. a) Schematic illustration of the conductivity mechanism of HMXTP hydrogel. b) The conductivity of different hydrogels. c) Comparison of real‐time ECG signals between HMXTP bioelectrodes and commercial electrodes at rest and after exercise. d) Comparison of SNR between HMXTP electrode and commercial electrode in resting and post‐exercise ECG signal acquisition. e) EMG signals measured by HMXTP bioelectrodes at different grip forces (30, 40, 50 lb). f) Comparison of EMG signal performance between the HMXTP bioelectrode and the commercial electrode under 24 kg grip force. g) Photos showing the influence of the commercial electrode and HMXTP bioelectrode on the skin after wearing for 6 h.

Most reported conductive hydrogels are prepared by physically blending conductive additives into hydrogel networks. However, this approach often results in heterogeneous dispersion and poorly connected conductive pathways, limiting conductivity and failing to meet the demands of high‐performance bioelectronics. In this study, an in situ polymerization strategy was employed, using MX‐T as a functional template to facilitate the assembly of PEDOT on its surface. This strategy significantly enhances the electrical conductivity of the hydrogel network. To investigate the influence of MX‐T‐P nanosheet content on the hydrogel's electrical properties, the conductivity of PAM and HMXTP hydrogels was characterized (**Figure** **4**a,b). The PAM hydrogel, as expected, was nearly non‐conductive. The HMXT5P exhibited relatively high conductivity, as displayed in Table S2 (Supporting Information), markedly exceeding that of MXene only or PEDOT: PSS‐based conductive hydrogels. This substantial enhancement demonstrates the effectiveness of MX‐T‐P in forming highly interconnected and efficient conductive pathways. The outstanding conductivity of the HMXTP hydrogel can be primarily attributed to the incorporation of tannic acid, which promotes multiple hydrogen‐bonding interactions that facilitate π─π stacking between PEDOT^+^ chains and the MXene surface (Figure 4a). This improvement arises from the TA modification layer, which strengthens the interfacial interactions between MXene and PEDOT, enabling their synergistic integration as dual electron‐conducting components and thereby improving charge carrier mobility. Moreover, the resulting MX‐T‐P nanosheets exhibit excellent aqueous dispersibility, allowing for their uniform distribution within the hydrogel matrix and the formation of continuous, efficient conductive pathways.

Additionally, the HMXTP hydrogels maintained stable conductivity during storage, with only a minor ≈5% decrease primarily due to partial water loss (Figure S17, Supporting Information). Moreover, negligible conductivity decay was observed after 100 loading‐unloading cycles at 100% strain, demonstrating excellent electrical stability under repeated mechanical deformation, with values remaining substantially higher than those of native biological tissues (0.3–0.7 S·m^−1^).^[^ ^51^ ^]^ Notably, this level of conductivity is sufficient to meet the requirements for high‐sensitivity electrophysiological signal monitoring.^[^ ^53^ ^]^ Given the intended use of HMXTP hydrogels for bioelectronics, their biocompatibility is of critical importance. As shown in Figure S18 (Supporting Information), predominant green fluorescence and negligible red fluorescence were observed, consistent with the control group. Additionally, the cytotoxic of hydrogels was further evaluated by F‐actin and DAPI nuclear staining to visualize the actin cytoskeleton and vinculin, respectively. The results revealed a well‐organized actin network with outstretched filopodia and lamellipodia (red), indicating that the cells exhibited uniform monolayer spreading, proliferation, and growth. To further assess in vivo biocompatibility (Figure S20, Supporting Information), histological analysis (H&E staining) demonstrated that the hydrogel had good biocompatibility and did not cause fibrotic reactions or inflammatory responses in the surrounding tissues after 14 days of implantation. Consequently, the integration of high conductivity, stretchability, self‐adhesion, and biocompatibility endows HMXTP hydrogels with strong potential as flexible, wearable sensors for health monitoring.

Electrophysiological signals, such as ECG, EMG, and EEG, provide critical insights into physiological activities and disease states. Among them, ECG signals, generated by the depolarization and repolarization of cardiac myocytes, are widely used in clinical diagnostics to detect conditions such as congenital heart defects, arrhythmias, and heart failure.^[^ ^54^, ^55^ ^]^ Stable and high‐quality recording of these weak bioelectrical signals is essential not only for clinical applications but also for the development of human‐machine interfaces. Given the stretchability, adhesion, and high conductivity of the HMXTP hydrogel, a self‐assembled biosignal acquisition system was constructed for real‐time ECG monitoring. The HMXTP hydrogel electrodes and commercial Ag/AgCl electrodes were simultaneously applied to the skin and connected to a real‐time computer‐based monitoring system. As shown in Figure S21 (Supporting Information), the HMXTP hydrogel exhibited more stable and clearly defined ECG waveforms than the commercial electrodes. To comprehensively evaluate the monitoring capability of the HMXTP conductive hydrogel under different physiological conditions, ECG signals were recorded from subjects both at rest and following physical activity. The HMXTP hydrogel electrodes consistently captured complete P‐QRS‐T waveforms in both states. At rest, a heart rate of 86 bpm was recorded, increasing to 107 bpm after physical activity, in line with physiological responses. Compared to commercial Ag/AgCl electrodes, the HMXTP electrode exhibited superior signal clarity and stability, with well‐defined signals maintained throughout the recording process (Figure 4c). Quantitative evaluation of signal quality was conducted via signal‐to‐noise ratio (SNR) analysis. At rest, the SNR of HMXTP hydrogel electrodes reached (24.07 ± 1.3) dB, outperforming Ag/AgCl electrodes at (20.17 ± 0.7) dB. After exercise, HMXTP hydrogel electrodes maintained a high SNR of (22.62 ± 0.2) dB, while the SNR of Ag/AgCl electrodes dropped significantly to (10.93 ± 1.8) dB (Figure 4d). The SNR decline for HMXTP hydrogel electrodes was only ≈6%, compared to ≈45% for Ag/AgCl electrodes.

To evaluate the suitability of HMXTP conductive hydrogels for EMG signal acquisition, a series of experiments was designed. As shown in Figure 4e, the HMXTP hydrogel electrodes clearly captured EMG signal variations correlated with grip strength, with increasing amplitude under higher force(30, 40, and 50 lb). For further validation, HMXTP hydrogels and Ag/AgCl electrodes were applied to the arm muscles using a dual‐channel system. During five repeated grips at ≈24 kg force, the HMXTP hydrogel electrodes recorded EMG signals with high clarity, sharp voltage fluctuations, and consistent tracking of muscular effort (Figure 4f). Furthermore, to evaluate long‐term wearability, the electrodes were continuously worn on human skin. As shown in Figure 4g, the Ag/AgCl electrodes caused visible skin indentation and redness, while the HMXTP hydrogel, owing to its tissue‐like softness, caused no irritation or adverse reaction, demonstrating excellent skin compatibility and wearing comfort. To evaluate the performance of the HMXTP bioelectrode under practical conditions, we assessed its signal transmission capability on sweaty skin. EMG recordings were compared between the HMXTP hydrogel electrodes and commercial electrodes after running. As shown in Figure S22 (Supporting Information), the commercial electrodes were nearly detached from the skin, resulting in substantial loss of EMG signal variation. In contrast, the HMXTP bioelectrode maintained stable adhesion and delivered high‐resolution EMG signals, demonstrating its reliable performance under sweating conditions.

**Figure 5:**
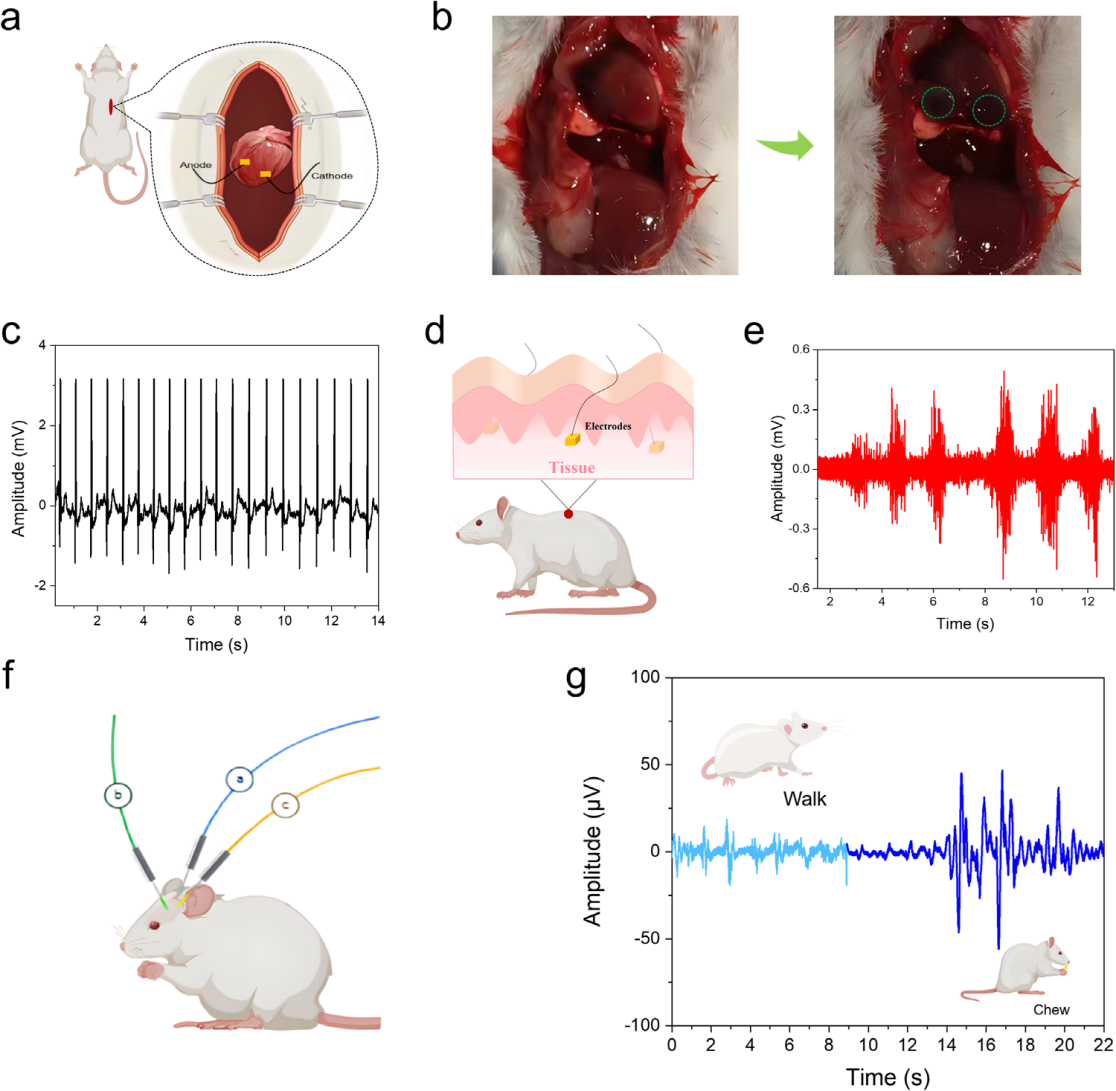
The in vivo bioelectrical signal detection of HMXTP conductive hydrogels. a) and b) Schematic illustration and photograph of an ECG recording. c) ECG signals acquired by HMXTP bioelectrodes. d) Schematic illustration of EMG recording. e) EMG signals acquired by HMXTP bioelectrodes. f) Schematic illustration of EEG recording. g) EEG signals collected by HMXTP bioelectrodes when the rat was walking or chewing.

Benefiting from its tissue‐matched mechanical properties, self‐adhesion, high conductivity, and excellent biocompatibility, the HMXTP conductive hydrogel offers a promising platform for establishing efficient communication between electronic devices and biological tissues. As proof of concept, a series of in vivo bioelectrical monitoring tests was designed (**Figure** **5**). As a proof of concept, HMXTP hydrogel electrodes were directly adhered to the rat heart (Figure 5b) to capture epicardial ECG signals. As shown in Figure 5c, the hydrogel recorded stable and complete ECG signals without baseline drift or high‐amplitude noise artifacts induced by cardiac motion. Moreover, their self‐adhesion caused no observable stress responses or arrhythmias. To further evaluate the application potential, the HMXTP conductive hydrogels were implanted subcutaneously on the rat's back for EMG signal acquisition, as schematically shown in Figure 5d. The hydrogels successfully captured muscle activity with high signal clarity (Figure 5e), indicating stable integration with the surrounding tissue. Furthermore, their use in EEG monitoring was demonstrated, as shown in Figure 5f. Real‐time EEG signals recorded during different behaviors, including walking and chewing, displayed clear and distinct waveforms (Figure 5g), demonstrating outstanding signal‐resolving capability.

**Figure 6:**
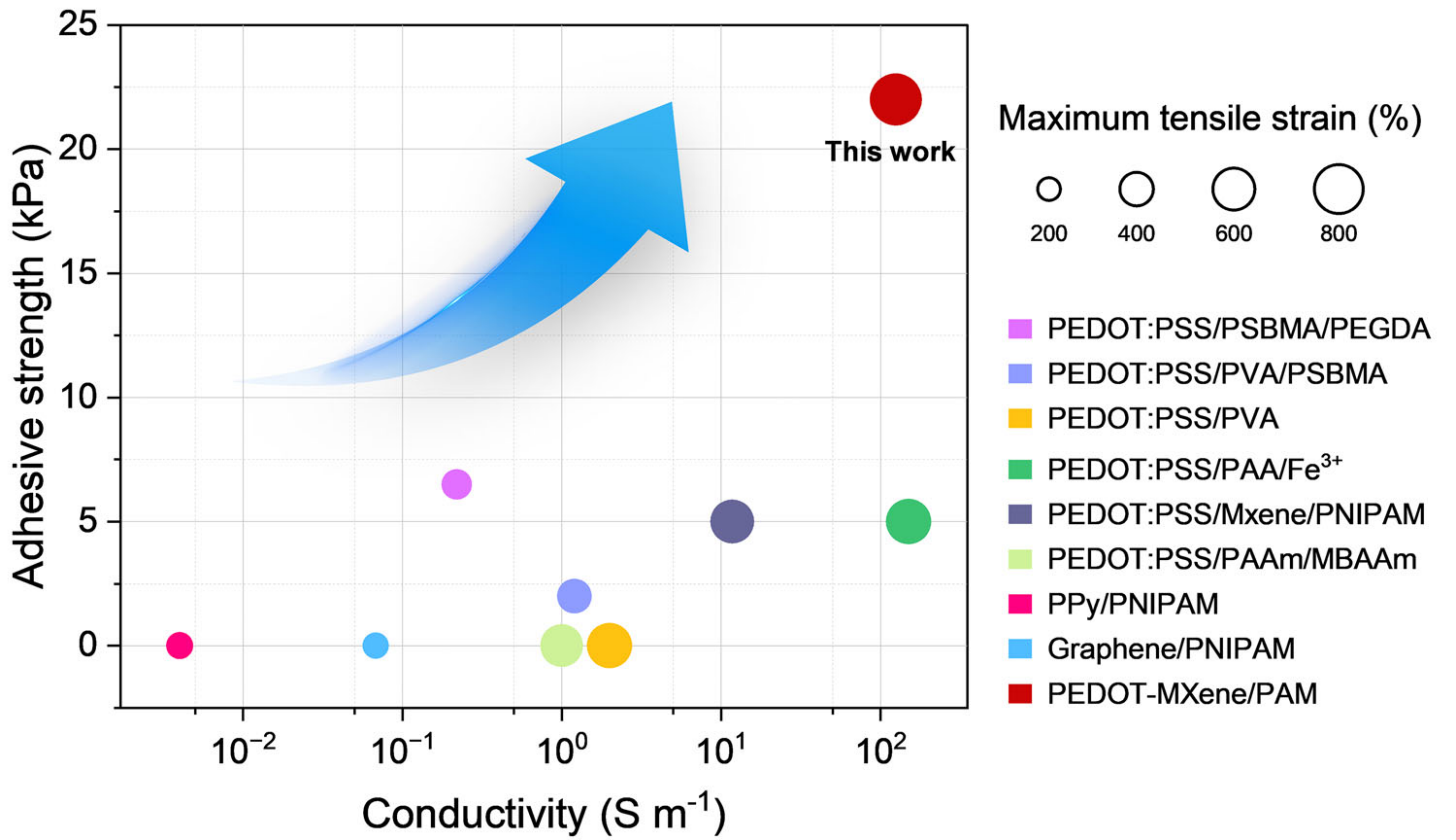
A comparative analysis of conductivity, maximum tensile strain, and adhesive strength between this work and recently reported conductive hydrogels.^[^ ^32^, ^56^, ^57^, ^58^, ^59^, ^60^, ^61^, ^62^, ^63^ ^]^

Collectively, these results confirm the broad application potential of HMXTP conductive hydrogels in electrophysiological signal monitoring. Their excellent mechanical stretchability and adhesiveness ensure stable contact with biological tissues, while their high conductivity guarantees accurate and reliable signal transmission. As illustrated in **Figure** **6**, the HMXTP hydrogel exhibits superior adhesion and conductivity compared to most reported PEDOT: PSS‐based hydrogels, thereby confirming the effectiveness of the proposed design strategy. The exceptional conductivity of the hydrogel is primarily attributed to two key factors: (i) TA modification enhances multiscale hydrogen‐bonding interactions between microscale conductive MXene sheets and nanoscale PEDOT chains, facilitating efficient charge transport; and (ii) the MX‐T‐P nanosheets possess excellent aqueous dispersibility, enabling their uniform integration into the hydrogel matrix and the formation of continuous conductive pathways. In addition to its outstanding electrical and adhesive properties, the hydrogel demonstrates remarkable mechanical stretchability, withstanding strains exceeding 800%, which is sufficient to accommodate the complex motions and deformations associated with human body movement. Together, these integrated properties underscore the potential of HMXTP hydrogels as a strong candidate for next‐generation bioelectronic applications.

## 3 Conclusion

In summary, this work proposes an effective strategy for the fabrication of PEDOT composite nanosheets by employing TA modified MXene as a functional template to direct the in situ polymerization of EDOT. The TA modification increases the density of surface active sites on the MXene surface, thereby enhancing interfacial interactions with EDOT. As a result, the obtained MX‐T‐P nanosheets exhibit excellent aqueous dispersibility and electrical conductivity (385 S·m^−1^), which can be attributed to the multiscale hydrogen bonding between the MXene nanosheets and the nanoscale PEDOT chains, enabling efficient charge transport. Benefiting from their excellent dispersion, MX‐T‐P nanosheets can be uniformly incorporated into the PAM hydrogel matrix, significantly enhancing its mechanical performance. The dynamic hydrogen bonding between the phenolic hydroxyl groups of MX‐T‐P and the amide groups in PAM serves as sacrificial bonds, effectively dissipating energy under strain and endowing the hydrogel with high stretchability (> 800%). Moreover, the integration of MX‐T‐P nanosheets acts as multifunctional fillers, enabling the hydrogel to simultaneously achieve high stretchability, strong adhesion (≈22 kPa), and high conductivity (≈125 S·m^−1^). These synergistic properties enable stable and high‐fidelity acquisition of electrophysiological signals, as demonstrated by reliable ECG and EMG recordings, highlighting the hydrogel's potential for wearable bioelectronic applications. Overall, this study not only addresses a persistent challenge in the development of high‐performance conductive polymer hydrogels but also provides a promising way for advancing next‐generation hydrogel‐based bioelectronic devices and applications.

## 4 Experimental Section

### Materials

Lithium fluoride, Ti_3_AlC_2_, hydrochloric acid, ammonium persulfate (APS), bis‐acrylamide (BIS), iron chloride hexahydrate, and 3,4‐ethylenedioxythiophene were purchased from Aldrich Co. Acrylamide, tannic acid, and N,N,N',N'‐tetramethylethylenediamine(TEMED) were purchased from Signa–Aldrich Co. All chemical reagents were used without further purification.

### Preparation of a Few‐Layered Ti_3_C_2_T_x_ MXene Dispersion

The Ti_3_C_2_T_x_ MXene aqueous dispersion was prepared following a previously reported method.^[^ ^64^ ^]^ Briefly, 1.98 g of LiF was dissolved in 30 mL of 6 m hydrochloric acid under stirring at room temperature for 5 min. Then, 3 g of Ti_3_AlC_2_ MAX phase powder was gradually added to the above solution over a period of 10 min. The mixture was stirred continuously at 40 °C for 45 h to selectively etch the Al layer. After etching, the mixture was washed repeatedly with deionized water and centrifuged until the pH of the supernatant reached ≈6.

### Preparation of MX‐T‐P Nanosheets

The MX‐T‐P nanosheets were prepared using the following procedure. First, the freshly prepared MXene solution was ultrasonicated in an ice bath for 60 min to ensure uniform dispersion. Then, tannic acid powder was gradually added under slow stirring until fully dissolved. The pH of the system was subsequently adjusted stepwise using a tris buffer solution. The mixture was stirred continuously at room temperature under light‐protected conditions for 24 h. This process yielded a homogeneous and stable MXene#TA dispersion.

The obtained MXene#TA dispersion was then subjected to an additional 30 min ultrasonication in an ice bath to ensure well‐dispersed, large‐sized sheet structures. Afterward, 50 mL of EDOT/ethanol solution was added to the reaction system and stirred vigorously for 30 min to achieve uniform dispersion of EDOT. Subsequently, a FeCl_3_ solution (0.5 g mL^−1^) was added dropwise into the MXene#TA dispersion under continuous stirring in an ice bath. The reaction was allowed to proceed for 24 h to promote the polymerization of EDOT and its successful assembly onto the MXene#TA substrate.

Finally, the product was collected by centrifugation and repeatedly washed with deionized water and ethanol to obtain the target MXene#TA‐PEDOT (MX‐T‐P) nanosheets. Depending on the mass ratio of EDOT to MXene#TA, the resulting products were designated as MX‐T‐1P, MX‐T‐3P, MX‐T‐5P, MX‐T‐7P, and MX‐T‐10P, respectively. The dispersion shown in Figure 2i, PEDOT, MXene, and MX‐T‐P were dispersed in deionized water at approximate concentrations of 1.4 mg mL^−1^.

### Preparation of HMXTP Hydrogels

The HMXTP hydrogels were synthesized using the following procedure. Briefly, acrylamide, APS, BIS, and various proportions of nanomaterials were added into 10 mL of deionized water and stirred continuously for 20 min until fully dissolved. Subsequently, TEMED was introduced into the mixture under stirring in an ice bath. Finally, the resulting prepolymer solution was poured into a mold, where polymerization occurred to form a hydrogel with a 3D network structure. HMXT5P hydrogel was selected for discussions and experiments, unless otherwise specified.

### Characterization

The morphology and energy dispersive spectroscopy (EDS) maps of freeze‐dried hydrogel were characterized by a Scanning electron microscope (SEM) on a Helios Nanolab 600i system (FEI). The crystalline structures were examined by X‐ray powder diffraction (XRD) with Ni‐filtered Cu Kα radiation. The chemical composition was analyzed by X‐ray photoelectron spectroscopy (XPS, ESCALab250‐XI, Thermo Scientific) with a monochromatic Al Kα source, and all binding energies were calibrated to the C 1s peak at 284.6 eV. Structural analysis of chemical bonds was further conducted using Fourier transform infrared (FTIR) spectroscopy (Nicolet, Thermo Scientific) equipped with an attenuated total reflectance (ATR) accessory. The tensile test was performed on a tensile tester (WDW3100, Changchun Kexin Co., China) at a constant deformation rate of 50 mm min^−1^.

### Electrical Characterization

The conductivity of the hydrogels (thickness = 1 mm) was measured by a two‐probe method^[^ ^65^ ^]^ via an electrochemical workstation (CHI 660C Instruments, USA). The conductivity was calculated as follows equation:

(1) σ=I/V×L/A

where V was the measured voltage; I was the current provided by the potential state; A (cm^2^) was the cross‐sectional area of the sample, and L was the distance between the two probes.

### Adhesion Energy Measurements

The lap‐shear tests were performed with a mechanical testing machine (100N load cell). All tests were conducted with a constant peeling speed of 50 mm min^−1^. Shear strength was calculated by dividing the maximum force by the adhesion area.

### In Vitro Biocompatibility of Hydrogel

The cytocompatibility of our hydrogels was evaluated using NIH3T3 cells (iCell Bioscience Inc., Shanghai, China; RRID: CVCL_L992). NIH3T3 cells were seeded in a 96‐well plate at a density of 6000 cells well^−1^ (n = 5 per each group), cultured in DMEM supplemented in a 5% CO_2_ incubator at 37 °C for 12h. After incubation for 72 h, 100 µL of CCK‐8 solution was added to each well and then incubated for another 2h at 37°C, respectively. The optical density value at 450 nm was measured using a microplate reader (SPARK 10M, TECAN, Switzerland).

In order to conduct the effect of hydrogel on the morphology and viability of the cells were evaluated with a live/dead kit. NIH3T3 cells were seeded in a 24‐well plate at a density of 60 000 cells well^−1^, cultured in DMEM supplemented in a 5% CO_2_ incubator at 37 °C for 12h, and then co‐cultured with the hydrogel extract corresponding to dilution extents in an incubator for 72 h under the same conditions as before. NIH3T3 cells were stained with Calcein‐AM and PI (dead) after co‐cultured with the hydrogels. The samples were observed using a fluorescent microscope (FV1200, Olympus, Japan).

### In Vivo Biocompatibility Evaluation

HMXTP hydrogel was implanted under the skin on the back of rats. Before implantation, HMXTP hydrogel was exposed to UV light for 2 h. Subsequently, a hydrogel was subcutaneously implanted in the left flank of a rat and then retrieved after 14 days. The harvested tissue was collected were fixed in a 10% neutral buffered formalin solution for 48 h, and was stained with hematoxylin and eosin for evaluating the biocompatibility of the hydrogels. The images of samples were observed using an optical microscope (Olympus, BX53) for subsequent histological analysis.

### Bioelectronic Applications of HMXTP Hydrogel

HMXTP hydrogels were employed as bioelectronic electrodes for recording EMG, ECG, and EEG signals, which were acquired using a multichannel physiological signal acquisition system. The biosignals were detected from the authors whom was informed and agreed to do the experiments prior to the study. No formal approval for the experiments involving human volunteers was required. Before the experiment, the volunteer (the first author) had been informed about the details of the human experiment and agreed to participate. All animal procedures were approved by the Animal Experimentation Ethics Committee of Research Selection Biotechnology (Hangzhou) Co., LTD (permission number SYXK (Zhejiang)2022‐0043), and all procedures were performed in strict accordance with the Guide for the Care and Use of Laboratory Animals and the Regulation of Animal Protection Committee.

## Conflict of Interest

The authors declare no conflict of interest.

## Author Contributions

R. G. W. and X. D. H. supervised the work. H. Q. S. carried out most experiments and wrote the manuscript. S. W. and F. Y. contributed to the manuscript editing. P. P. W., L. B., and M.Y.T. revised the manuscript. All authors have approved the final version of the manuscript.

## Supporting information



Supporting Information

## Data Availability

The data that support the findings of this study are available from the corresponding author upon reasonable request.
