# Supplementary material for: Highly Stretchable, Adhesive, and Conductive PEDOT Nanocomposite Hydrogels for High‐Performance Flexible Bioelectronics
Source: Adv Sci (Weinh). 2025 Oct 7;12(48):e13487. doi: 10.1002/advs.202513487 (PMC12752577; doi:10.1002/advs.202513487)
Supplement: Supplementary file 1 — Supporting Information [file ADVS-12-e13487-s001.pdf]

Supporting Information

Highly Stretchable, Adhesive, and Conductive PEDOT Nanocomposite Hydrogels for High-Performance Flexible Bioelectronics

*Huiqi Sun, Sai Wang *, Peipei Wang, Ling Bai, Mingyi Tan*, Fan Yang *, Rongguo Wang *, and Xiaodong He*

H. Sun, P. Wang, L. Bai, M. Tan, F. Yang, R. Wang, and X. He
National Key Laboratory of Science and Technology on Advanced Composites in Special Environments, Harbin Institute of Technology, Harbin 150000, China
E-mail: wrg@hit.edu.cn (Prof. R. Wang)

S. Wang
School of Mechatronic Engineering, Shenzhen Polytechnic, Shenzhen 518055, China
E-mail: wangsai@szpt.edu.cn (Dr. S. Wang)

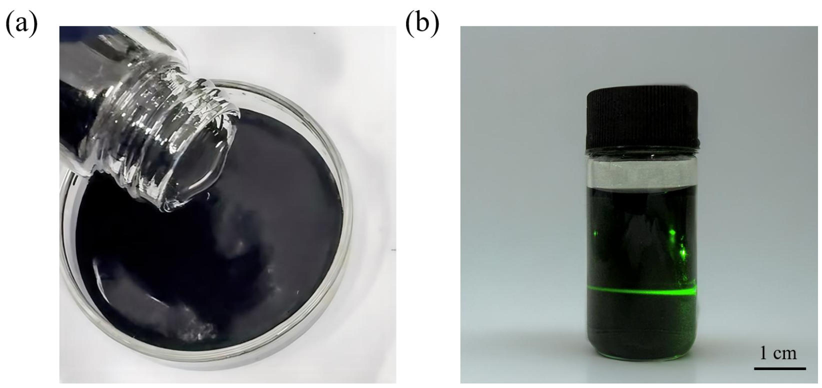

Figure S1. Macroscopic photograph of MXene. (a) Image of the MXene colloidal dispersion; (b) Observation of the Tyndall effect in the MXene dispersion.

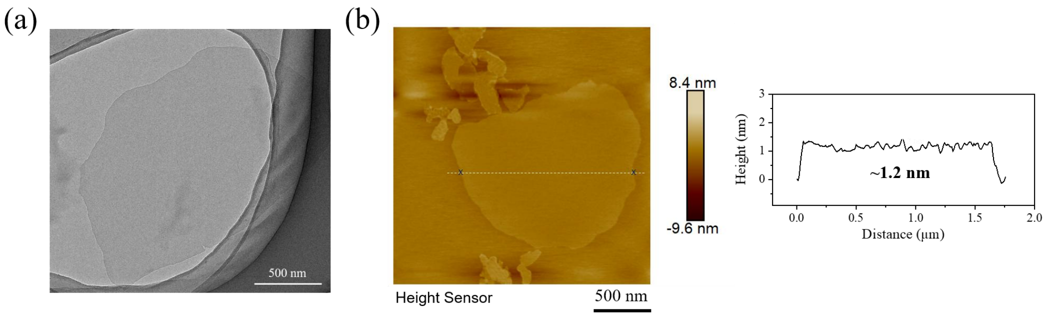

Figure S2. Image of large-size single-layer MXene. (a) TEM image; (b) AFM topography and corresponding flake thickness.

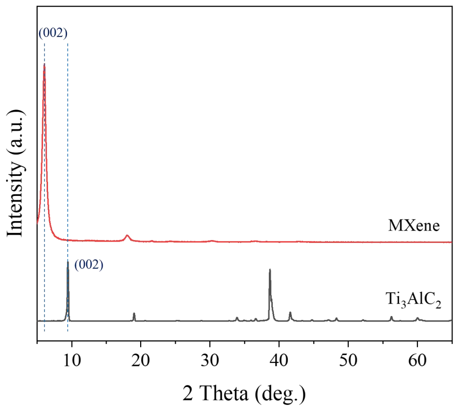

Figure S3. XRD patterns of Ti_3AlC_2 and MXene nanosheets.

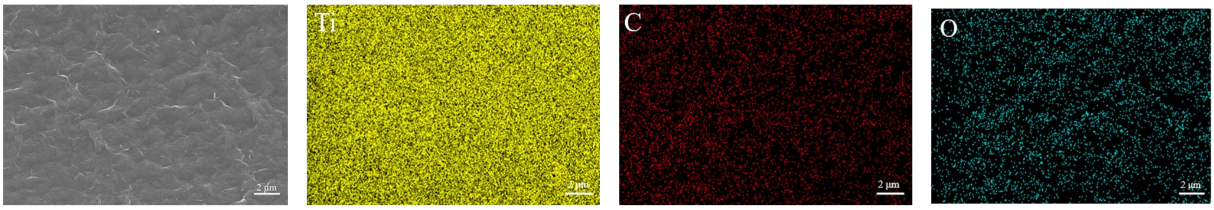

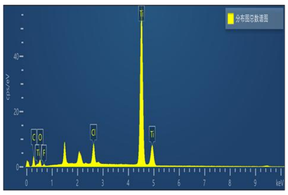

Element	Wt%	At%
Ti	79.04	55.32
C	8.19	22.85
O	7.33	15.36
F	1.62	2.86
Cl	3.82	3.61
Total	100	100

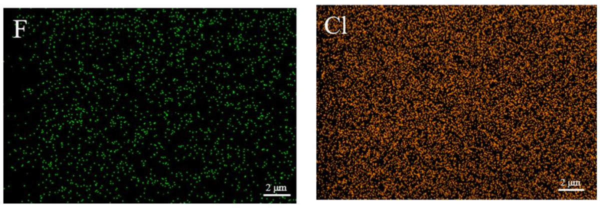

Figure S4. SEM patterns and elemental distribution of MXene nanosheets.

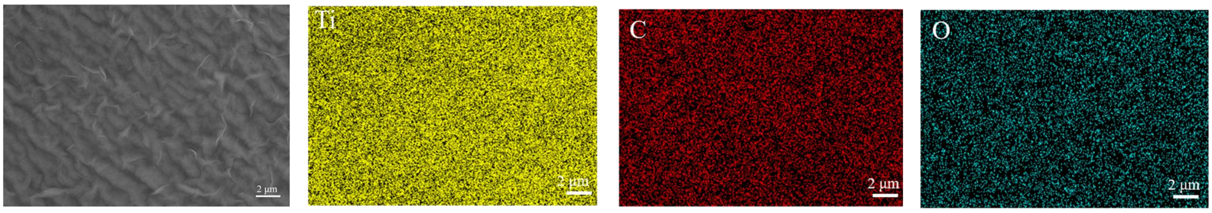

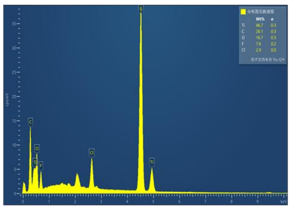

Element	Wt%	At%
Ti	46.7	20.87
C	26.07	46.45
O	16.73	22.38
F	7.58	8.54
Cl	2.92	1.76
Total	100	100

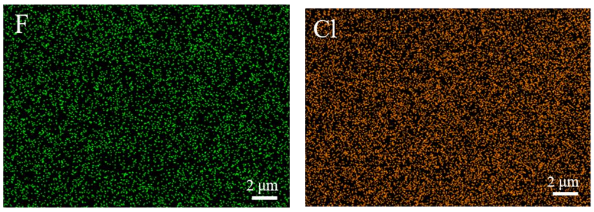

Figure S5. SEM patterns and elemental distribution of MXene#TA nanosheets.

.

Table S1 Energy dispersive x-ray spectra of MXene and MXene#TA atomic percentage of major elements (at%)

Materials	Ti	C	O
MXene	51.28±4.19	23.08±1.66	17.44±1.85
MXene#TA	23.53±4.96	43.58±4.46	22.50±0.23

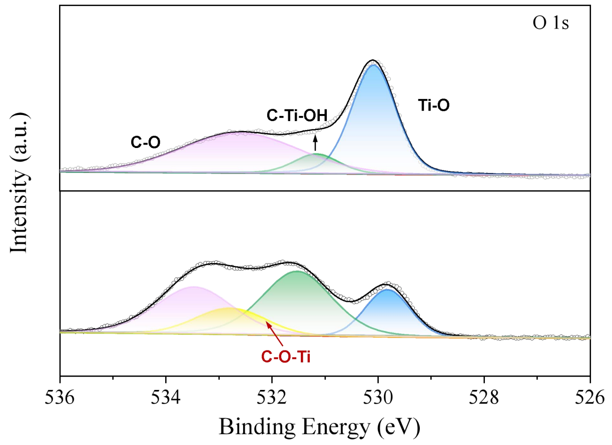

Figure S6. XPS spectra of O 1s for MXene and MXene#TA.

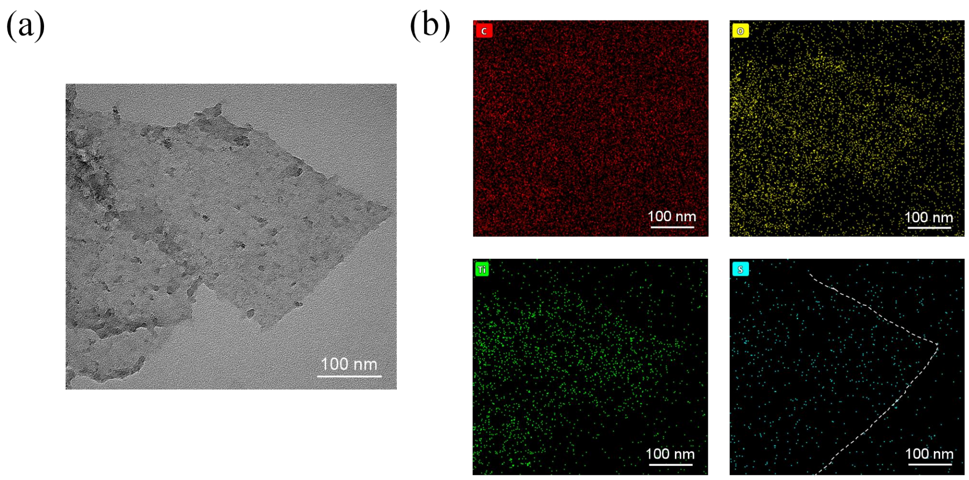

Figure S7. TEM image (a) and corresponding elemental distribution maps of MX-T-P nanosheets (b), showing carbon (C, red), oxygen (O, yellow), titanium (Ti, green), and sulfur (S, blue).

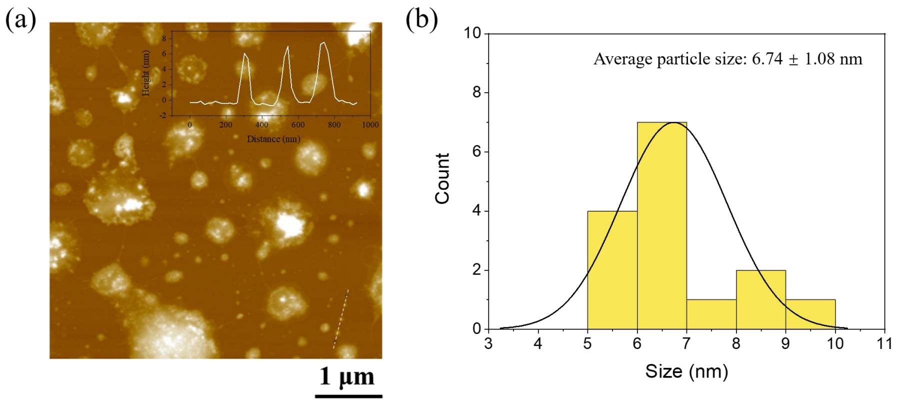

Figure S8. AFM characterization of PEDOT: (a) surface morphology and (b) particle size distribution.

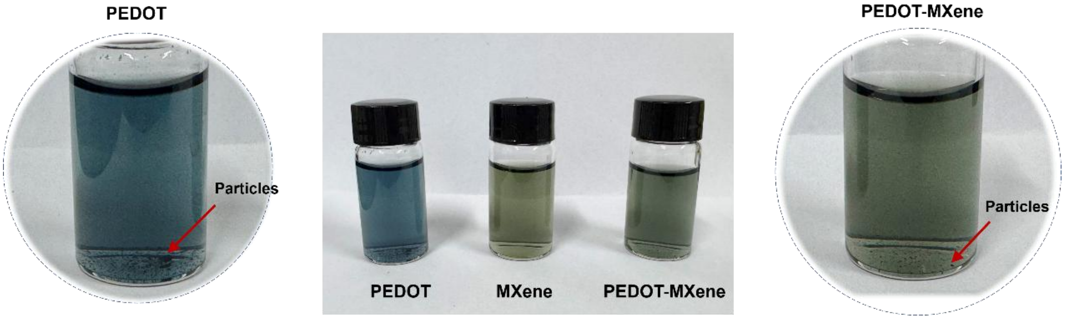

Figure S9. Dispersion of PEDOT, MXene, and PEDOT-MXene nanomaterials in water.

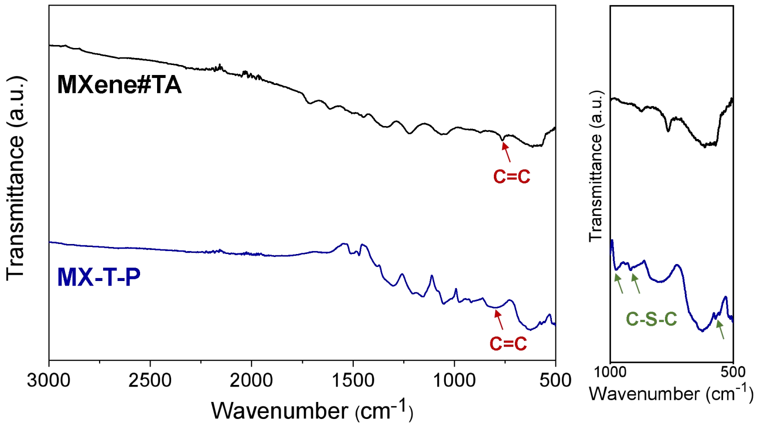

Figure S10. Fourier transform infrared spectroscopy (FTIR) spectrum of MXene#TA and MX-T-P.

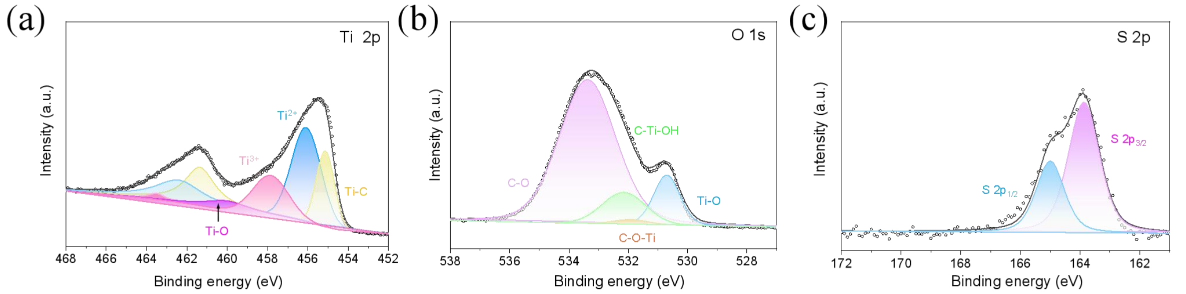

Figure S11. High-resolution XPS spectra of Ti 2p (a), O 1s (b), and S 2p (c) for MX-T-P nanosheets.

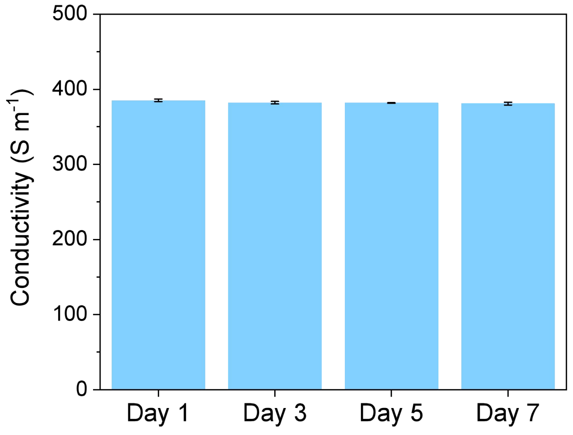

Figure S12. Electrical conductivity of MX-T-3P nanosheets after 7 days of storage (25 °C, 30%RH).

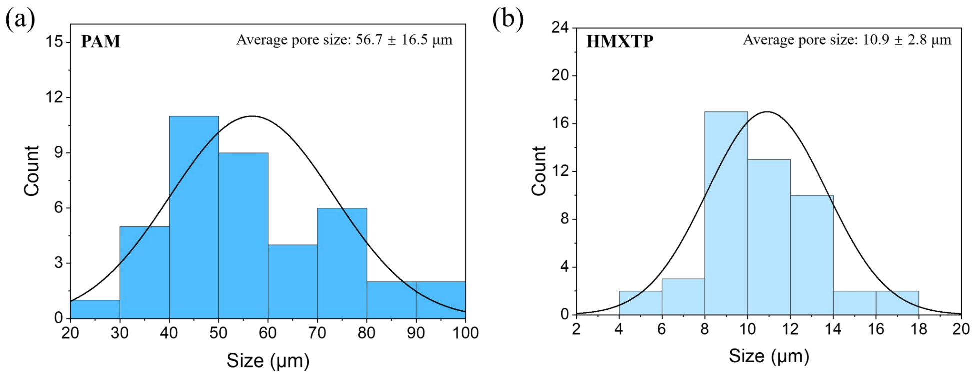

Figure S13. Pore size distribution of (a) PAM hydrogel and (b) HMXTP hydrogel.

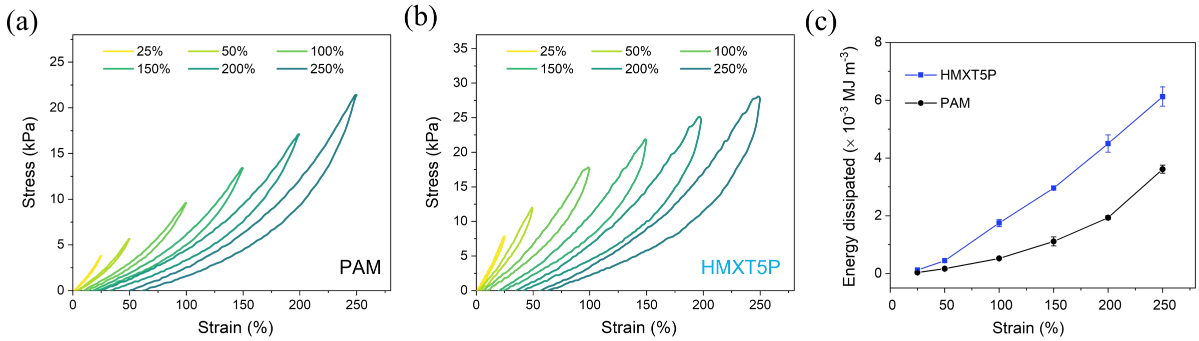

Figure S14. Cyclic tensile loading–unloading curves of (a) PAM hydrogel and (b) HMXT5P hydrogel; (c) Energy dissipation of PAM and HMXT5P hydrogels at different strains, calculated from the corresponding loading-unloading curves.

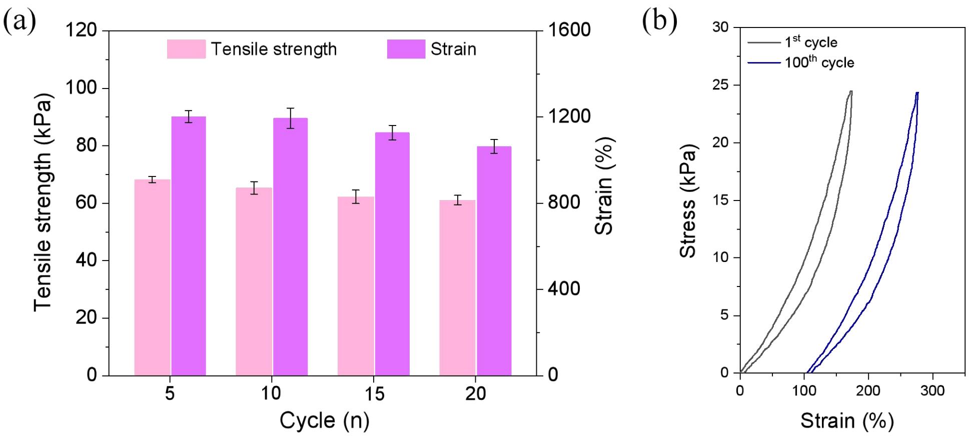

Figure S15. Mechanical properties of HMXT5P hydrogel: (a) repeated stretching cycles at 100% strain; (b) consecutive loading–unloading tensile cycles under 180% strain for 100 cycles, with curves horizontally offset for clarity.

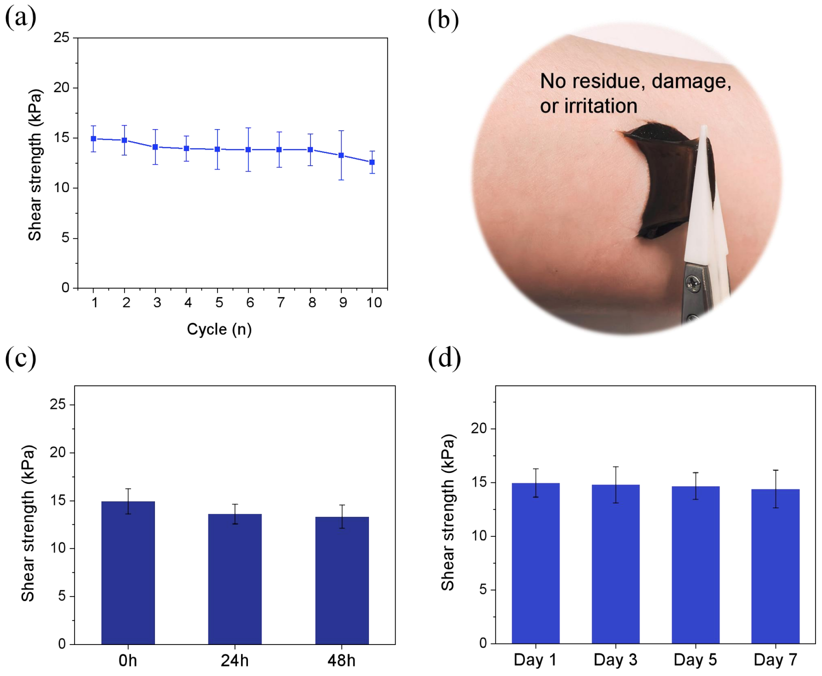

Figure S16. Adhesive properties of HMXT5P hydrogel. (a) Repeatable adhesive strength of the hydrogel on skin tissue; (b) Repeated adhesion of the hydrogel on human skin, which can be completely removed without residue; (c) Adhesive strength of the hydrogel after immersing in PBS at 37 °C for 48 h; (d) Adhesive strength of the hydrogel after 7 days of storage at 4 °C.

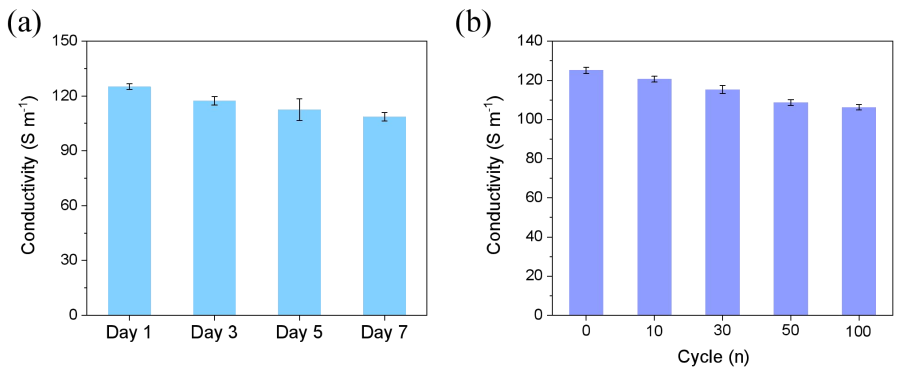

Figure S17. Conductivity of HMXT5P hydrogel. (a) Conductivity of the hydrogel after 7 days of storage at 4 °C; (b) Conductivity of the hydrogel after repeated stretching cycles at 100% strain.

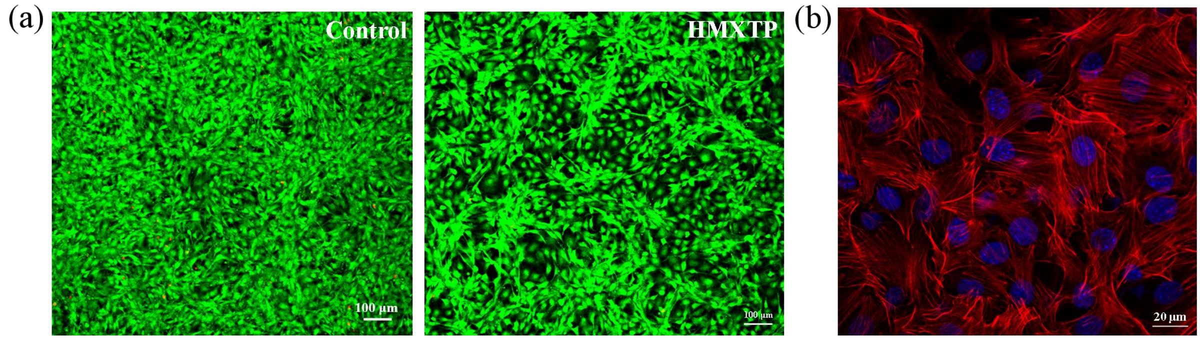

Figure S18. Biocompatibility of HMXTP hydrogels *in vitro*. (a) Live/dead viability staining; (b) Fluorescent staining of F-actin and nuclei with DAPI.

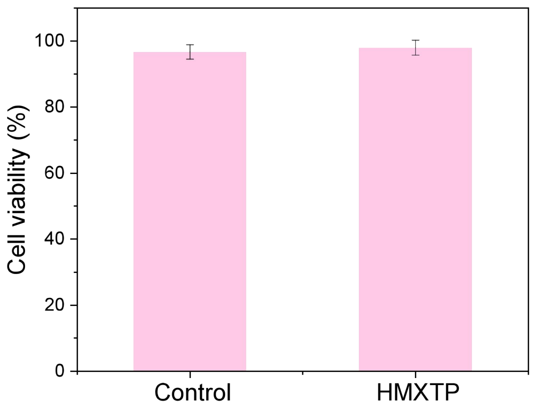

Figure S19. In vitro cell viability of NIH3T3 cells based on CCK-8 assay after 72h of culture.

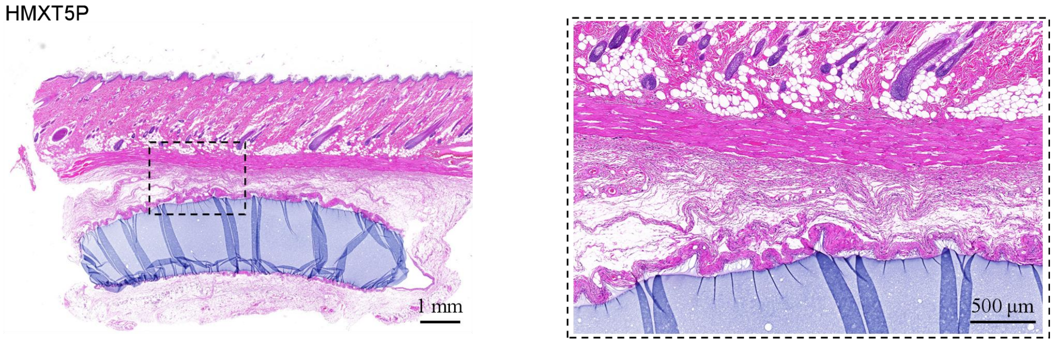

Figure S20. H&E-stained histological images of HMXT5P hydrogels 14 days after subcutaneous implantation.

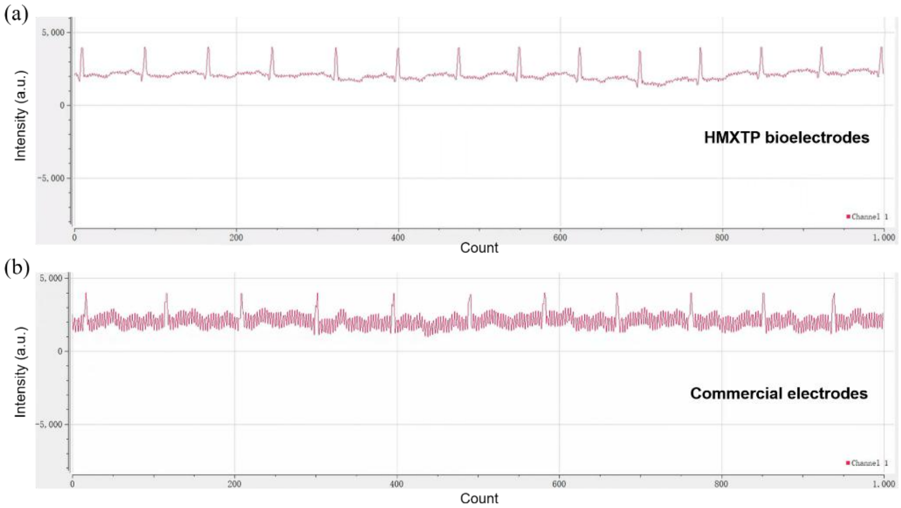

Figure S21. Real-time ECG signal comparison between HMXTP bioelectrodes and commercial electrodes in computer-based monitoring systems. (a) HMXTP bioelectrodes; (b) Commercial electrodes.

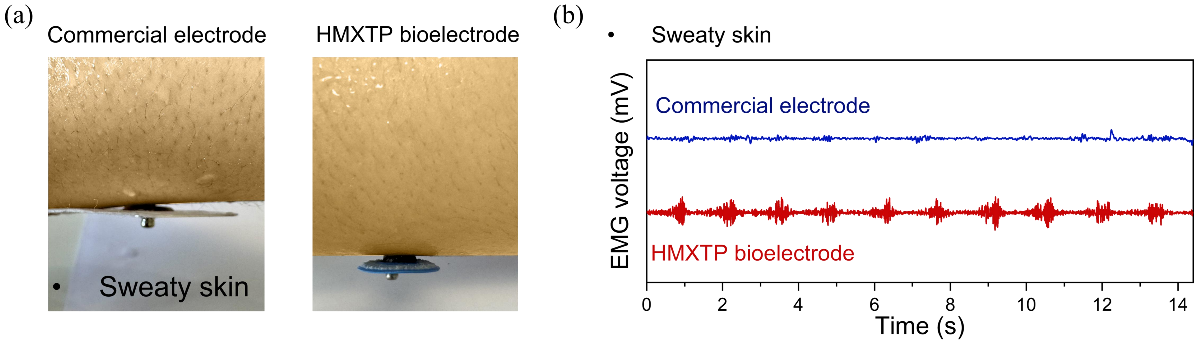

Figure S22. Detection of EMG signals on sweaty skin. (a) Photographs of a commercial electrode and the HMXTP bioelectrode applied to sweaty skin; (b) Corresponding EMG signals recorded from the two electrodes.

Table S2. A comparative analysis of the properties of the conductive hydrogel materials presented in this work and those reported in previous literature.

Materials (Major components)	Mechanical property		Conductivity (S m^{-1})	Adhesion Properties (kPa)	SNR (dB)	Ref
	Elastic modulus (kPa)	Strain (%)				
Au/TPU	500	1500	-	Non-adhesiveness	17.8	[1]
GelMA/PDA/CNT	≈72	＜100	2.5	3.5	17	[2]
MXene/AgNP/Alg-PBA	-	100-200	0.003	-	18	[3]
PEDOT:PSS-PSBMA-PEGDA	-	120-390	0.22	6.5	21	[4]
PEDOT:PSS-PAM-PDA	≈2.7	850	5.57	46.5	30	[5]
PEDOT:PSS-PVA-PSBMA	≈65	349	1.2	2	16	[6]
**PEDOT-MXene/PAM**	**≈80-100**	**＞800**	**125**	**~22**	**24**	**This work**

References

[1] S. Pan, F. Zhang, P. Cai, M. Wang, K. He, Y. Luo, Z. Li, G. Chen, S. Ji, Z. Liu, X.J. Loh, X. Chen, Mechanically Interlocked Hydrogel–Elastomer Hybrids for On-Skin Electronics, Adv. Funct. Mater. 30(29) (2020) 1909540. https://doi.org/https://doi.org/10.1002/adfm.201909540.

[2] H. Tang, Y. Li, B. Chen, X. Chen, Y. Han, M. Guo, H.-q. Xia, R. Song, X. Zhang, J. Zhou, In Situ Forming Epidermal Bioelectronics for Daily Monitoring and Comprehensive Exercise, ACS Nano 16(11) (2022) 17931-17947. https://doi.org/10.1021/acsnano.2c03414.

[3] M. Li, Y. Zhang, L. Lian, K. Liu, M. Lu, Y. Chen, L. Zhang, X. Zhang, P. Wan, Flexible Accelerated-Wound-Healing Antibacterial MXene-Based Epidermic Sensor for Intelligent Wearable Human-Machine Interaction, Adv. Funct. Mater. 32(47) (2022) 2208141. https://doi.org/https://doi.org/10.1002/adfm.202208141.

[4] X. Huang, C. Chen, X. Ma, T. Zhu, W. Ma, Q. Jin, R. Du, Y. Cai, M. Zhang, D. Kong, M. Wang, J.a. Ren, Q. Zhang, X. Jia, In Situ Forming Dual-Conductive Hydrogels Enable Conformal, Self-Adhesive and Antibacterial Epidermal Electrodes, Adv. Funct. Mater. 33(38) (2023) 2302846. https://doi.org/https://doi.org/10.1002/adfm.202302846.

[5] R. Wan, J. Yu, Z. Quan, H. Ma, J. Li, F. Tian, W. Wang, Y. Sun, J. Liu, D. Gao, J. Xu, B. Lu, A reusable, healable, and biocompatible PEDOT:PSS hydrogel-based electrical bioadhesive interface for high-resolution electromyography monitoring and time–frequency analysis, Chem. Eng. J. 490 (2024) 151454. https://doi.org/https://doi.org/10.1016/j.cej.2024.151454.

[6] J. Yu, R. Wan, F. Tian, J. Cao, W. Wang, Q. Liu, H. Yang, J. Liu, X. Liu, T. Lin, J. Xu, B. Lu, 3D Printing of Robust High-Performance Conducting Polymer Hydrogel-Based Electrical Bioadhesive Interface for Soft Bioelectronics, Small 20(19) (2024) 2308778. https://doi.org/https://doi.org/10.1002/smll.202308778.
